# Military Personnel with Chronic Symptoms Following Blast Traumatic Brain Injury Have Differential Expression of Neuronal Recovery and Epidermal Growth Factor Receptor Genes

**DOI:** 10.3389/fneur.2014.00198

**Published:** 2014-10-09

**Authors:** Morgan Heinzelmann, Swarnalatha Y. Reddy, Louis M. French, Dan Wang, Hyunhwa Lee, Taura Barr, Tristin Baxter, Vincent Mysliwiec, Jessica Gill

**Affiliations:** ^1^National Institute of Nursing Research, National Institutes of Health, Bethesda, MD, USA; ^2^Center for Neuroscience and Regenerative Medicine, Bethesda, MD, USA; ^3^Defense and Veterans Brain Injury Center, Walter Reed National Military Medical Center, Bethesda, MD, USA; ^4^West Virginia University Health Sciences Center, Morgantown, WV, USA; ^5^Sleep Medicine Clinic, Madigan Army Medical Center, Tacoma, WA, USA

**Keywords:** traumatic brain injury, military, post-concussive disorder, gene-expression

## Abstract

**Objective:** Approximately one-quarter of military personnel who deployed to combat stations sustained one or more blast-related, closed-head injuries. Blast injuries result from the detonation of an explosive device. The mechanisms associated with blast exposure that give rise to traumatic brain injury (TBI), and place military personnel at high risk for chronic symptoms of post-concussive disorder (PCD), post-traumatic stress disorder (PTSD), and depression are not elucidated.

**Methods:** To investigate the mechanisms of persistent blast-related symptoms, we examined expression profiles of transcripts across the genome to determine the role of gene activity in chronic symptoms following blast-TBI. Active duty military personnel with (1) a medical record of a blast-TBI that occurred during deployment (*n* = 19) were compared to control participants without TBI (*n* = 17). Controls were matched to cases on demographic factors including age, gender, and race, and also in diagnoses of sleep disturbance, and symptoms of PTSD and depression. Due to the high number of PCD symptoms in the TBI+ group, we did not match on this variable. Using expression profiles of transcripts in microarray platform in peripheral samples of whole blood, significantly differentially expressed gene lists were generated. Statistical threshold is based on criteria of 1.5 magnitude fold-change (up or down) and *p*-values with multiple test correction (false discovery rate <0.05).

**Results:** There were 34 transcripts in 29 genes that were differentially regulated in blast-TBI participants compared to controls. Up-regulated genes included epithelial cell transforming sequence and zinc finger proteins, which are necessary for astrocyte differentiation following injury. Tensin-1, which has been implicated in neuronal recovery in pre-clinical TBI models, was down-regulated in blast-TBI participants. Protein ubiquitination genes, such as epidermal growth factor receptor, were also down-regulated and identified as the central regulators in the gene network determined by interaction pathway analysis.

**Conclusion:** In this study, we identified a gene-expression pathway of delayed neuronal recovery in military personnel a blast-TBI and chronic symptoms. Future work is needed to determine if therapeutic agents that regulate these pathways may provide novel treatments for chronic blast-TBI-related symptoms.

## Introduction

Improvised explosive devices (IEDs) have induced blast traumatic brain injuries (TBI) in approximately one-quarter of military personnel deployed to combat stations in Operations Enduring Freedom and Iraqi Freedom (1). In addition to the blast wave, blast events often involve a blunt-force component, placing individuals at risk for alterations in consciousness, disruptions in memory, and headaches from both mechanisms of the blast-TBI (2, 3). Following deployment these military personnel are at high risk for on-going neurological and psychological symptoms, including post-concussive disorder (PCD), post-traumatic stress disorder (PTSD), and depression (4, 5). Our understanding of a complete and precise mechanism of blast-related pathology is limited, resulting in an inability to determine military personnel at risk for these chronic disorders, and to inform interventions to mitigate these risks.

Clinical studies report differential gene expression following TBI; however, these studies do not include blast-TBI, and most use post-mortem, neuronal tissue samples obtained from severe TBI patients (6–10). Studies that use imaging techniques report cortical thinning in the left superior temporal and frontal gyri (11) and altered neurocircuitry (12) in military personnel with a history of blast-TBI and chronic symptoms. These current studies are limited because they could neither examine the biological processes that contributed to neuronal compromise nor did they determine the impact of other variables that may affect gene expression or morphology of the brain, including comorbid PTSD, depression, and chronic pain. These considerations are essential, as not-determining the impact of these complexities can result in an inability to determine what the consequences are from the blast itself (13). Although there are advantages of pre-clinical models of blast, there are also challenges in translating findings that include differential injury mechanisms in the laboratory, limiting application to clinical patients including military personnel (14).

Although pre-clinical models are not in agreement in the preferential models of blast-TBI, there is definitive evidence linking blast-TBIs to neuronal function changes (15). Blast-TBIs result in cerebral edema and vasospasm, which contribute to global acute neuronal compromise through an influx of immune cells and inflammatory processes (16, 17). Within 24 h of blast-TBI, an up-regulation of glial fibrillary acidic protein, vimentin, and complement component 1 is observed (18), as well as an activation of microglia (19), which are key to TBI recovery as they propagate inflammation to neighboring neurons, providing vital protection. In traditional TBI, microglia communicates with peripheral immune cells to modulate immune activities (20). Specifically, microglia interacts with peripheral immune cells, resulting in a gene-expression signature in the peripheral blood cells that may be informative of central recovery activities (21). This assertion has been recently supported in a pre-clinical study that reports similar micro-RNA changes in the cerebral spinal fluid and serum following a blast, suggesting that peripheral samples provide insights into neuronal changes following blast (22).

Therefore, we suggest that central neuronal recovery mechanisms may be detected in the periphery through whole blood samples obtained from military personnel with a history of blast-TBI who are seeking care for chronic symptoms. To further investigate this postulation, we enrolled military personnel with medically documented blast-TBI, as well as controls without TBI. Instruments and a clinical evaluation were undertaken to determine current symptoms of PCD, PTSD, depression, and sleep disturbance. Differentially expressed genes related to blast-TBI were obtained by comparing military personnel with blast-TBI to matched controls without TBI using a whole-genome approach that eliminates any biased selection of biological targets. We addressed the possible impact of PTSD, depression, and sleep disturbance on gene-expression through group matching, on diagnoses of sleep disturbance, and symptom severity of PTSD and depression. This approach may provide a minimally invasive opportunity to investigate the gene-expression pathways that contribute to blast-TBI pathology in military personnel. We expect this line of research to ultimately inform pharmacological agents to better treat military personnel with chronic symptoms and deficits following a blast-TBI.

## Materials and Methods

### Clinical methods

This study was an observational assessment of U.S. military personnel at the Madigan Army Medical Center who had been deployed within the previous 18 months. Exclusion criteria included (1) no history of drug or alcohol abuse in the previous year and (2) no current medical condition that required substantial treatments (cancer, diabetes, HIV, autoimmune disorders), or a severe psychiatric condition (i.e., schizophrenia or bipolar disorder). Subjects with neurological disorders other than headaches, e.g., multiple sclerosis, seizure disorders, and a history of stroke, were excluded. Subjects with blast-TBI were identified by having a blast-TBI documented in their medical record. Blast-TBI cases did not require a pre-specified severity of PCD symptoms. Controls neither have a medical history of any TBI in their records nor report a TBI when asked. Controls were matched to blast-cases as much as possible on critical variables that would have influenced gene-expression including the following: age, gender, race/ethnicity, and diagnoses of sleep disorder, and symptom severity of PTSD and depression. The medical record was used to determine the number and type of prescribed medications that military personnel were prescribed. This study was approved by the institutional review board at the Madigan Army Medical Center, and informed consent was obtained from each individual prior to any baseline measurements.

#### Determination of TBI

The case history of TBI was extracted from medical records of the 117 military personnel enrolled in a larger observational study. Clinical information obtained includes the following: type of injury, diagnosis of TBI that includes severity, loss of consciousness, and time since the TBI. To be a blast-TBI case, the participant must also have reported sustaining a TBI through administration of the warrior administered retrospective casualty assessment tool (WARCAT) (23). A negative response to the WARCAT was necessary to be in the control group. This tool obtains data on possible war-related TBI injuries and post-deployment injuries, and whether they were injured from mechanisms commonly associated with TBI while deployed, whether any injuries resulted in an altered mental status indicative of TBI, and/or whether specific somatic and neuropsychiatric symptoms commonly associated with mild TBI occurred after the injury (immediately post-injury and/or post-deployment). The somatic and neuropsychiatric symptoms were not used to make the diagnosis of TBI. The diagnosis of mild TBI was made in accordance with American Congress of Rehabilitation Medicine mild TBI criteria, which requires an injury event followed by a loss of consciousness or alteration of mental state and was consistent with criteria for TBI delineated by the Department of Defense and Department of Veterans Affairs.

#### Determination of PCD, PTSD, and depression symptoms

The neurobehavioral symptom inventory (NSI) was used to measure PCD symptom severity. The NSI is a 22-item measure designed to evaluate self-reported post-concussion symptoms (e.g., headache, balance, nausea, etc.). The NSI requires the test taker to rate the presence/severity of each symptom on a 5-point scale (none, mild, moderate, severe, very severe). A total score is obtained by summing the ratings for the 22 items (range = 0–88). The NSI has a high-internal consistency (total alpha = 0.95; subscale alpha = 0.88–0.92) and reliability (*r* = 0.88–0.93) (24). This instrument was administered to all participants.

Symptoms of PTSD were assessed by the PTSD checklist military version (PCL-M), resulting in a score between 0 and 80, with higher numbers indicating the greatest severity (25). The quick inventory of depressive symptomatology (QIDS) was used to measure total symptoms of depression, resulting in a score of 0–27 (26). Chronic pain was evaluated by the bodily pain score from the short form (36) health survey (SF-36). We used this in conjunction with the medical record to determine a diagnosis of pain, by either a score lower than 70 for military personnel with a pain diagnosis in their electronic medical record or a low-bodily pain subscale score (<30) on the SF-36 (27).

### Laboratory methods

#### RNA sample preparation

Blood samples were collected in PAXgene blood RNA tubes and processed with PAXgene™ Blood RNA Kits (PreAnalytiX, Qiagen) for RNA extraction according to the manufacturer’s instruction. Quality and quantity of extracted RNA were evaluated with the NanoDrop DN-1000 spectrophotometer (Thermo Fisher Scientific, Wilmington, DE, USA) and the Agilent Bioanalyzer 2100 eukaryotic total RNA Nano assay (Agilent Technologies, Inc., Santa Clara, CA, USA). The 260/280 ratio ranged 2.03–2.34 and the RNA integrity numbers (RIN) were >7.0 in all samples.

#### Microarray gene-expression profiling

Purified RNAs (100 ng) were amplified, fluorescently labeled, and hybridized to Affymetrix GeneChip Human Gene U133 Plus 2.0 Arrays (Affymetrix, Inc., Santa Clara, CA, USA), which contain more than 54,000 probes representing thousands of specific gene coding regions. After thorough washing, the raw data were obtained by laser scanning imaging. All RNA preparation and transcriptome assays were undertaken in the NINR laboratory based on manufacturer’s protocols.

#### Data analysis

Descriptive statistics for all demographic and clinical variables were calculated using SPSS Statistics (IBM SPSS Inc., Chicago, IL, USA) (Tables 1 and 2). Comparisons were made between the groups using *t*-tests. *A priori p*-values <0.05 were considered significant.

**Table 1:** **Demographics and clinical characteristics for blast-TBI + PCD (*N* = 19) and control (*N* = 17) groups**.

	TBI + PCD (*N* = 19)	Control (*N* = 17)	Significance
Mean age in years (SD)	32.00 (9.04)	30.94 (7.48)	*F*_1,35_ = 0.14, *p* = 0.72
Mean BMI (SD)	29.30 (3.76)	31.65 (4.82)	*F*_1,35_ = 2.03, *p* = 0.16
Gender, % (No)			N/A
Male	100.0% (19)	100.0% (17)	
Race, % (No)			χ^2^ = 4.22, *p* = 0.52
Caucasian	73.8% (14)	58.8% (10)
Mixed	15.8% (3)	11.8% (2)	
All other	10.4% (2)	19.4% (5)	
Rank, % (No)			χ^2^ = 0.97, *p* = 0.62
Lower enlisted	57.9% (11)	58.8% (10)	
Senior NCO	36.8% (7)	35.3% (6)	
Officer	5.2% (1)	5.9% (1)	
Sleep diagnoses, % (No)			χ^2^ = 2.79, *p* = 0.43
OSA	10.5% (2)	5.9% (1)	
Insomnia	31.6% (8)	23.5% (4)	
OSA + insomnia	31.5% (6)	58.8% (10)	
None	15.8% (3)	11.8% (2)	
Depression severity (QIDS)	9.6 (5.1)	8.7 (4.8)	*F*_1,35_ = 2.42, *p* = 0.10
PTSD severity (PCL-M)	32.05 (18.8)	29.90 (15.1)	*F*_1,35_ = 2.31, *p* = 0.14
Prescribed medications, % yes (no)	36.8% (6)	41.2% (7)	χ^2^ = 1.51, *p* = 0.51
SSRI	15.8% (3)	11.8% (2)	
Prazosin	21.1% (4)	17.6% (3)	
Anti-hypertensive	15.8% (3)	23.5% (4)	

*TBI, traumatic brain injury; PCD, post-concussive disorder; SD, standard deviation; no, number; NCO, non-commissioned officer; BMI, body-mass index; OSA, obstructive sleep apnea; QIDS, quick inventory of depressive symptomatology; PTSD, post-traumatic stress disorder; PCL-M, PTSD checklist military version; SSRI, selective serotonin reuptake inhibitor*.

**Table 2:** **Neurobehavioral symptom inventory for blast-TBI + PCD (*N* = 19 and control (*N* = 17) groups**.

	TBI + PCD (*N* = 19)	Control (*N* = 17)	Significance
*Forgetfulness*	*3.19 (0.75)*	*1.71 (1.16)*	*F_1,35_* = *18.72, p* < *0.001*
*Concentration*	*2.69 (0.70)*	*1.29 (1.16)*	*F_1,35_* = *17.13, p* < *0.001*
*Slowed thinking*	*2.37 (0.89)*	*1.06 (1.30)*	*F_1,35_* = *11.44, p* = *0.002*
*Dizziness*	*1.19 (0.83)*	*0.41 (0.51)*	*F_1,35_* = *10.56, p* = *0.003*
*Headache*	*2.31 (1.08)*	*1.12 (1.05)*	*F_1,35_* = *10.36, p* = *0.003*
*Hearing problems*	*2.06 (1.18)*	*0.88 (1.22)*	*F_1,35_* = *7.96, p* = *0.008*
*Sleep difficulty*	*3.37 (0.72)*	*2.59 (0.87)*	*F_1,35_* = *7.96, p* = *0.008*
*Nausea*	*0.94 (1.06)*	*0.18 (0.39)*	*F_1,35_* = *7.63, p* = *0.010*
*Decision making*	*2.25 (1.07)*	*1.24 (1.15)*	*F_1,35_* = *6.91, p* = *0.013*
*Irritability*	*2.81 (0.66)*	*1.88 (1.32)*	*F_1,35_* = *6.46, p* = *0.016*
*Balance*	*1.19 (0.91)*	*0.53 (0.62)*	*F_1,35_* = *5.93, p* = *0.021*
*Numbness*	*2.06 (1.57)*	*0.82 (1.38)*	*F_1,35_* = *5.82, p* = *0.022*
*Coordination*	*1.38 (0.81)*	*0.65 (0.93)*	*F_1,35_* = *5.73, p* = *0.023*
*Frustration*	*2.44 (0.96)*	*1.53 (1.23)*	*F_1,35_* = *5.52, p* = *0.025*
*Light sensitivity*	*1.31 (1.08)*	*0.59 (0.87)*	*F_1,35_* = *4.54, p* = *0.041*
*Taste/smell changes*	*0.81 (0.98)*	*0.18 (0.73)*	*F_1,35_* = *4.51, p* = *0.042*
*Noise sensitivity*	*1.50 (1.21)*	*0.65 (1.12)*	*F_1,35_* = *4.44, p* = *0.043*
Vision problems	1.31 (1.14)	0.59 (0.94)	*F*_1,35_ = 3.99, *p* = 0.054
Anxiety	2.75 (1.24)	1.82 (1.51)	*F*_1,35_ = 3.69, *p* = 0.064
Depression	2.00 (1.03)	1.35 (1.27)	*F*_1,35_ = 2.55, *p* = 0.120
Appetite changes	1.56 (1.32)	0.88 (1.22)	*F*_1,35_ = 2.38, *p* = 0.133
Fatigue	2.75 (1.00)	2.47 (2.50)	*F*_1,35_ = 0.17, *p* = 0.680

*TBI, traumatic brain injury; PCD, post-concussive disorder*.

*Italic font: significant group difference. NSI scale ranges from 0 to 4 for each item, with a 0 indicating that the symptom is not present*.

Microarray data were analyzed for a total of 36 participants for gene-expression profiles using Partek Genomics Suite software (Partek Inc., St. Louis, MO, USA). The probe-level robust multichip average background correction, quantile normalization, log_2_ transformation, and probe-set summarization were performed on gene-expression intensity values. Multi-way analysis of variance (ANOVA) mixed models were constructed for differential expressions for both the blast-TBI + PCD (*N* = 19) and control (*N* = 17) groups. Since samples were processed on different dates, batch correction was applied. Pair-wise comparisons were made by setting the contrast between blast-TBI and control groups. Significant differentially expressed gene lists were generated based on criteria of 1.5 magnitude fold-change (up or down), and *p*-values corrected for false discovery rate (FDR < 0.05) using the step-up Benjamini–Hochberg method.

Interactive pathway analysis (IPA) (Qiagen Ingenuity Systems, Redwood City, CA, USA) was performed on differentially expressed genes to identify gene interactions and networks associated with biological functions.

Clinical variables were compared using ANOVA models for continuous variables, and chi-square for categorical data. Although our data were small in sample size, it met the assumptions of the tests we used, including ANOVA. Bonferroni corrections were used to adjust for multiple comparisons.

## Results

### Demographic and clinical characteristics

The demographic and clinical characteristics of the 36 participants used in this analysis are described in Table 1. The blast-TBI group (*N* = 19) was group matched to a control group (*N* = 17) to ensure that the groups did not differ on any demographic [age, body-mass index (BMI), gender, race, and military rank] or clinical characteristics (depression, and PTSD symptom severity and sleep diagnoses). There was no difference in the total number of military personnel taking any prescribed medication, or the type of medications, the most common being serotonin reuptake inhibitors (SSRIs), anti-hypertensive, and prazosin. The mean ages of the blast-TBI and control groups were 32.0 and 30.9 years, respectively. The sample was male, primarily Caucasian, and demonstrated high rates of comorbid symptoms of sleep disorders, depression, and PTSD. The blast-TBI group reported a far greater severity of PCD symptoms compared to controls.

All 19 participants in the TBI group experienced a blast injury, and 84.25% had a loss of consciousness following the blast-TBI. By medical diagnosis in theater, approximately 78.95% had a mild blast-TBI, and 21.05% of the sample was diagnosed with a moderate blast-TBI. In this sample, a brain contusion was reported to have occurred at the time of the blast in 36.84% of the sample, and all were determined to be mild in severity. More than half of the sample had more than one blast-TBI (52.6%), and approximately one-third had (38.8%) three or more blast-TBIs. The time since TBI varied, with 3–6 months, 6–12 months, and more than 12 months having elapsed for 15.8, 31.6, and 52.7% of the group, respectively.

The breakdown of the 22 components of the NSI for both the blast group and control group are shown in Table 2. Scores on 17 of the 22 components were significantly different between the two groups, based on a *p*-value of 0.05, and adjustment for multiple comparisons.

### Differential gene expression in TBI

**Figure 1:**
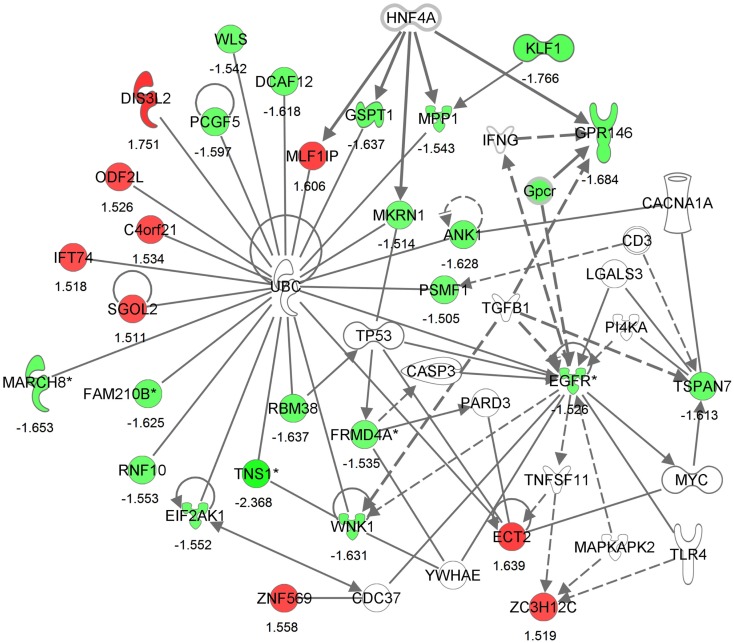
**Generated molecular network based on differential expression between traumatic brain injury (TBI) and control groups using ingenuity knowledge database**. Coloring is based on the expression values of the genes (fold changes shown), down-regulation in green and, up-regulation in red. Genes with no coloring are added from ingenuity knowledge database. Direct and indirect relationships are shown by solid and dashed lines, respectively. The arrow indicates specific directionality of interactions. Genes with an asterisk indicate that multiple identifiers (probe-sets) map to the gene in the molecular network.

Differential expression of transcripts between blast-TBI and control groups resulted in multiple down-regulated (Table 3) and up-regulated genes (Table 4). Notable down-regulated genes are membrane-associated ring finger (C3HC4) 8, E3 ubiquitin protein ligase (*MARCH8*, −1.6123 fold-change), tensin-1 (*TNS1*, −2.368 fold-change), tripartite motif containing 58 (*TRIM58*, −1.918), Kruppel-like factor 1 (*KLF1*, −1.766), WNK lysine deficient protein kinase 1 (*WNK1*, −1.630), ankyrin1 (*ANK1*, −1.628), and epidermal growth factor receptor (*EGFR*, −1.526). Differentially expressed gene pathways are shown in the network figure (Figure 1), generated from the ingenuity knowledge database. Figure 1 indicates that the majority of the genes relate to ubiquitin C (UBC), and that EGFR is related to multiple gene-networks.

**Table 3:** **Significantly down-regulated genes comparing blast-TBI + PCD (*N* = 19) to control (*N* = 17) groups**.

Probe-set ID	Gene symbol	Gene title	*p*-Value	Fold-change
221748	TNS1	Tensin-1	0.00062	−2.3682
215047	TRIM58	Tripartite motif containing 58	0.00012	−1.9188
210504	KLF1	Kruppel-like factor 1 (erythroid)	0.00094	−1.7663
221246	TNS1	Tensin-1	0.00061	−1.6963
228770	GPR146///LOC100505551///LOC100505568	G protein-coupled receptor 146///uncharacterized LOC100505551///uncharacterized LOC	0.00029	−1.684
221824	8-Mar	Membrane-associated ring finger (C3HC4) 8, E3 ubiquitin protein ligase	0.0001	−1.6534
201912	GSPT1	G1 to S phase transition 1	0.00057	−1.6369
212430	RBM38	RNA binding motif protein 38	0.00092	−1.6368
1555068	WNK1	WNK lysine deficient protein kinase 1	0.00017	−1.6307
218863	TNS1	Tensin-1	0.00066	−1.6286
205389	ANK1	Ankyrin 1, erythrocytic	0.00088	−1.6284
224690	FAM210B	Family with sequence similarity 210, member B	0.00054	−1.6254
224789	DCAF12	DDB1 and CUL4 associated factor 12	0.00019	−1.618
202242	TSPAN7	Tetraspanin 7	0.00015	−1.6131
231933	March8	Membrane-associated ring finger (C3HC4) 8, E3 ubiquitin protein ligase	0.00065	−1.6123
224693	FAM210B	Family with sequence similarity 210, member B	0.0008	−1.6016
227935	PCGF5	Polycomb group ring finger 5	0.00077	−1.5973
207801	RNF10	Ring finger protein 10	0.00057	−1.5533
217736	EIF2AK1	Eukaryotic translation initiation factor 2-alpha kinase 1	0.00035	−1.5522
202974	MPP1	Membrane protein, palmitoylated 1, 55 kDa	0.00079	−1.5427
221958	WLS	Wntless homolog (*Drosophila*)	0.00085	−1.5417
225167	FRMD4A	FERM domain containing 4A	0.00037	−1.5352
1565484	EGFR	Epidermal growth factor receptor	8.01E-06	−1.5261
201285	MKRN1	Makorin ring finger protein 1	0.00033	−1.5139
225168	FRMD4A	FERM domain containing 4A	0.00065	−1.5086
1565483	EGFR	Epidermal growth factor receptor	4.11E-07	−1.5053
235993	PSMF1	Proteasome (prosome, macropain) inhibitor subunit 1 (PI31)	0.00026	−1.505

*Microarray differentially expressed genes between groups that passed FDR (5%)*.

*TBI, traumatic brain injury; FDR, false discovery rate*.

**Table 4:** **Significantly up-regulated genes comparing blast-TBI + PCD (*N* = 19) to control (*N* = 17) groups**.

Probe-set ID	Gene symbol	Gene title	*p*-Value	Fold-change
242539	DIS3L2	DIS3 mitotic control homolog (*S. cerevisiae*)-like 2	0.000341	1.7507
219787	ECT2	Epithelial cell transforming sequence 2 oncogene	0.000281	1.6386
218883	MLF1IP	MLF1 interacting protein	8.53E-05	1.6065
1553696	ZNF569	Zinc finger protein 569	0.000737	1.5581
228859	C4orf21	Chromosome 4 open reading frame 21	0.000252	1.5335
231909	ODF2L	Outer dense fiber of sperm tails 2-like	0.000284	1.5258
231899	ZC3H12C	Zinc finger CCCH-type containing 12C	0.000174	1.5192
219174	IFT74	Intraflagellar transport 74 homolog (*Chlamydomonas*)	0.000763	1.5179
230165	SGOL2	Shugoshin-like 2 (*S. pombe*)	1.47E-05	1.5107

*Microarray differentially expressed genes between groups that passed FDR (5%)*.

*TBI, traumatic brain injury; FDR, false discovery rate*.

#### Sub-group analyses

We separated subjects with moderate TBI from mild, and also did a subsequent analysis on TBIs within the last 12 months compared to subjects with a TBI more than 12 months ago. Lastly, we grouped subjects by multiple blast-TBIs, compared to subjects with only one blast-TBI. None of these analyses yielded a significant finding over the 1.5-fold-threshold, with the adjustment for multiple comparisons.

## Discussion

To our knowledge this is the first study to report differential gene expression in the peripheral blood of military personnel with a history of blast-TBI during deployment compared to matched controls. Here, we report that differential expression of genes involved in the secondary injury processes may contribute to the long-term symptoms of PCD that are common among this group. These findings are significant because the genes we report to be differentially expressed are related to neuronal recovery following TBI, suggesting for the first time that a peripheral sample of blood may be used to better understand central processes in a sample of military personnel who have chronic blast-TBI related symptoms.

Specifically, our finding of a reduction in the membrane-associated ring finger (C3HC4) 8, E3 ubiquitin protein ligase (*MARCH8*) as well as tripartite motif containing 58 (TRIM58) in military personnel with blast-TBI suggest that adequate functionality of the ubiquitin proteasome is essential for recovery from blast-TBI. Protein ubiquitination protects neurons from the detrimental impact of accumulating reactive oxygen, nitrogen species, and free release of zinc initiates (28) by initiating the removal of oxidized or misfolded proteins that result following injury (18). In fact, higher concentrations of the protein encoded by UBC-terminal hydrolase-L1 is one of the primary proteomic biomarkers of acute TBI (29–31), with high concentrations being related to the onset of chronic symptoms in pediatric patients (30).

Pre-clinical models link blast-TBIs to the activation of the autonomous nervous system and the neuroendocrine–immune system, suggesting that neuronal pathology results from the over-activation of multiple biological systems (32). For example, blast-TBIs result in increases in inflammatory cytokines and chemokines, as well as orexin A, and neuropilin-2 release (33). Pre-clinical models also link blast to an increase in UCH-L1 in blood and CSF, with CSF elevations lasting for 14 days, as well as increases in other putative biomarkers of brain injury glial fibrillary acid protein, and neuron-specific enolase (34). Minimization of the ubiquitin pathway following a blast-TBI, by inducing hypothermic conditions, resulted in less UCH-L1 activity following blunt force and a reduction of neuronal and glial damage (35). Therefore, additional studies are needed to determine how the ubiquitin pathway relates to neuronal damage following blast, and if it may be a pharmacological target to promote neuronal recovery from blast.

Therefore, reduction in the expression of this gene suggests that this process is down-regulated and may contribute to chronic neurological symptoms and impairments in military personnel with a blast-TBI. This assertion is further supported by pre-clinical models that link down-regulation in protein ubiquitination genes to poor neuronal repair (36), greater neuronal loss (37, 38), and less neurodegeneration (39). A previous study has linked increased zinc accumulations to reduced function of the ubiquitin pathway in cultured neurons (40). Additional studies that include acute biomarkers and determine changes in gene-expression over time are needed to determine the role of protein ubiquitination in blast-TBI recovery, as this line of research may inform pharmacological agents to promote recovery.

We also report reduced expression of TNS1, a gene that is pivotal to neuronal recovery following a TBI, which works in coordination with protein ubiquitination genes. Thus, our finding of a reduction in TNS1 in conjunction with reductions in protein ubiquitination genes leads us to question if a down-regulation in these genes may compromise neuronal recovery following blast-TBI, resulting in chronic neurological symptoms and deficits. Increases in the activity of TNS1 are linked to a risk for the onset of cancer and allergy induced asthma (41, 42). In a recent study, TBIs have been associated with an increased risk for brain cancer (43), suggesting that this TNS1 activity may increase morbidity risks following TBI. Therefore, additional studies are needed to validate our finding and to determine how TNS1 may relate to blast-TBI and chronic neurological symptoms.

Axonal injury and degeneration, whether primary or secondary, contribute to the morbidity and mortality risks following TBI, which is shaped in part by the EGFR pathway through the activation of astrocytes. Astrocytes promote neuronal survival through potentiation of collateral synapses, migration of neuronal progenitor cells, and differentiation of glial progenitor cells following TBI (44, 45). EGFR is also a peripheral precursor for vascular endothelial growth factor (VEGF), which plays a role in angiogenesis and has been shown to be neuroprotective following TBI in pre-clinical models (46–48). Thus, EGFR is similar to protein ubiquitination, down-regulation, and may occur following blast-TBI, and place military personnel at greater risk for poor neuronal repair and insufficient regeneration, resulting in the development of chronic symptoms and deficits. Future studies that utilize biomarkers collected sooner following the blast-TBI in larger and more representative samples may determine this relationship.

Although many of the genes that were significantly different in the blast-TBI group compared to controls related to neuronal compromise, a number of genes were not. One such gene was up-regulation of MLF1 interacting protein, a gene that suppresses cancer cell progression (49), as well the gene for outer dense fiber of sperm tails 2-like (ODF2L), which relates to defective spermatozoa (50). In addition, we also did not observe alterations in genes linked to blast-TBI in pre-clinical models, including inflammation (33), and putative biomarkers of brain injury, including glial fibrillary acid protein and neuron-specific enolase (34). It may be that since many participants in our sample population had a TBI that occurred over a year prior to enrollment, other non-related processes may have occurred, possibly contributing to the identification of non-expected candidates.

Our findings are limited by the cross-sectional nature of the study in a relatively small sample of military personnel. Additional larger studies are required to confirm our findings. Additionally, our design did not allow for randomization. To combat this limitation, we matched controls to blast-TBI participants on diagnoses of sleep disturbance and symptom severity of PTSD and depression. Although this allowed for isolation of gene expression changes more related to blast-TBI, the heterogeneity of symptoms of PCD and times since the blast event were issues that we were not able to address with the current design. In addition, without a third comparison group with a TBI not related to blast, we are unable to be certain that our findings are related to blast-TBI. We were limited in not including a clinician administered diagnostic tool to determine diagnoses of PTSD and depression, which is an issue in this group of patients who often present with complex symptoms that may be psychosomatic in nature. We report high rates of comorbidity in PCD, PTSD, and depression symptoms, as well as perceived health declines (51), and greater psychosomatic illness severity (52). Future larger studies that allow for comparison of differing symptom profiles of PCD and other comorbid symptoms will provide insights into both the shared and unique gene-expression pathways that underlie complex clinical presentations following blast-TBI.

Taken together, the identification of down-regulation in protein ubiquitination and mediation of this pathway in gene expression, as well as down-regulation of EGFR and TNS1, suggest involvement of these gene-expression pathways in chronic TBI pathology. Both of these gene-pathways protect neurons from secondary injuries following TBI and are highly specific to neurons (53, 54), and suggest that secondary injury or repair cascades can be detected in a peripheral sample of blood from military personnel with blast-related injuries even when injuries are not acute. Future studies are necessary to determine how variation in these gene-expression pathways may contribute to the heterogeneity in recovery from blast-TBI, as well as how manipulation of these pathways through the administration of pharmacological agents may promote recovery.

## Conflict of Interest Statement

No author has any conflicts of interest to disclose. The opinions and assertions in this manuscript are those of the authors and do not necessarily represent those of the Department of the Army, Department of Defense, U.S. Government, or the Center for Neuroscience and Regenerative Medicine.

## Abbreviations

ANOVA, analysis of variance; BMI, body-mass index; DSM-IV, diagnostic and statistical manual of mental disorders, 4th ed; EGFR, epidermal growth factor receptor; FDR, false discovery rate; IPA, interactive pathway analysis; MARCH8, membrane-associated ring finger (C3HC4) 8, E3 ubiquitin protein ligase; NSI, neurobehavioral symptom inventory; OSA, obstructive sleep apnea; PCD, post-concussive disorder; PCL-M, PTSD checklist military version; PTEN, phosphatase and tensin homolog; PTSD, post-traumatic stress disorder; QIDS, quick inventory of depressive symptomatology; SCID, structured clinical interview for DSM-III-R; SF-36, short form (36) health survey; TBI, traumatic brain injury; TNS1, tensin-1; VEGF, vascular endothelial growth factor; WARCAT, warrior administered retrospective casualty assessment tool; ZC3H12C, zinc finger CCCH-type containing 12C.

## References

[1] Owens BD Kragh JF Jr Wenke JC Macaitis J Wade CE Holcomb JB Combat wounds in operation Iraqi freedom and operation enduring freedom. J Trauma (2008) 64(2):295–9 10.1097/TA.0b013e318163b875 18301189

[2] DePalma RG Burris DG Champion HR Hodgson MJ Blast injuries. N Engl J Med (2005) 352(13):1335–42 10.1056/NEJMra042083 15800229

[3] Warden DL French LM Shupenko L Fargus J Riedy G Erickson ME Case report of a soldier with primary blast brain injury. Neuroimage (2009) 47(Suppl 2):T152–3 10.1016/j.neuroimage.2009.01.060 19457364

[4] Seelig AD Jacobson IG Smith B Hooper TI Boyko EJ Gackstetter GD Sleep patterns before, during, and after deployment to Iraq and Afghanistan. Sleep (2010) 33(12):1615–22 21120123 10.1093/sleep/33.12.1615 PMC2982731

[5] Tsai JC Neurological and neurobehavioral sequelae of obstructive sleep apnea. Neurorehabilitation (2010) 26(1):85–94 10.3233/NRE-2010-0538 20130357

[6] Zhou YW Zhang YG Deng WN [A primary study on the ARP-SRP gene expression profiling of brain injury by cDNA microarray]. Fa Yi Xue Za Zhi (2002) 18(3):146–9 10.1529/biophysj.106.100776 12608292

[7] Michael DB Byers DM Irwin LN Gene expression following traumatic brain injury in humans: analysis by microarray. J Clin Neurosci (2005) 12(3):284–90 10.1016/j.jocn.2004.11.003 15851083

[8] Kukacka J Vajtr D Huska D Prusa R Houstava L Samal F Blood metallothionein, neuron specific enolase, and protein S100B in patients with traumatic brain injury. Neuro Endocrinol Lett (2006) 27(Suppl 2):116–20 17159794

[9] Frugier T Crombie D Conquest A Tjhong F Taylor C Kulkarni T Modulation of LPA receptor expression in the human brain following neurotrauma. Cell Mol Neurobiol (2011) 31(4):569–77 10.1007/s10571-011-9650-0 21234797 PMC11498475

[10] Frugier T Conquest A McLean C Currie P Moses D Goldshmit Y Expression and activation of EphA4 in the human brain after traumatic injury. J Neuropathol Exp Neurol (2012) 71(3):242–50 10.1097/NEN.0b013e3182496149 22318127

[11] Tate DF York GE Reid MW Cooper DB Jones L Robin DA Preliminary findings of cortical thickness abnormalities in blast injured service members and their relationship to clinical findings. Brain Imaging Behav (2014) 8(1):102–9 10.1007/s11682-013-9257-9 24100952 PMC4714342

[12] Yeh PH Wang B Oakes TR French LM Pan H Graner J Postconcussional disorder and PTSD symptoms of military-related traumatic brain injury associated with compromised neurocircuitry. Hum Brain Mapp (2014) 35(6):2652–73 10.1002/hbm.22358 24038816 PMC6869078

[13] Rosenfeld JV McFarlane AC Bragge P Armonda RA Grimes JB Ling GS Blast-related traumatic brain injury. Lancet Neurol (2013) 12(9):882–93 10.1016/S1474-4422(13)70161-3 23884075

[14] Panzer MB Wood GW Bass CR Scaling in neurotrauma: how do we apply animal experiments to people? Exp Neurol (2014) 261C:120–6 10.1016/j.expneurol.2014.07.002 25035134

[15] Effgen GB Vogel EW III Lynch KA Lobel A Hue CD Meaney DF Isolated primary blast alters neuronal function with minimal cell death in organotypic hippocampal slice cultures. J Neurotrauma (2014) 31(13):1202–10 10.1089/neu.2013.3227 24558968

[16] Ling G Bandak F Armonda R Grant G Ecklund J Explosive blast neurotrauma. J Neurotrauma (2009) 26(6):815–25 10.1089/neu.2007.0484 19397423

[17] Cho HJ Sajja VS Vandevord PJ Lee YW Blast induces oxidative stress, inflammation, neuronal loss and subsequent short-term memory impairment in rats. Neuroscience (2013) 253:9–20 10.1016/j.neuroscience.2013.08.037 23999126

[18] Kochanek PM Dixon CE Shellington DK Shin SS Bayır H Jackson EK Screening of biochemical and molecular mechanisms of secondary injury and repair in the brain after experimental blast-induced traumatic brain injury in rats. J Neurotrauma (2013) 30(11):920–37 10.1089/neu.2013.2862 23496248 PMC5586163

[19] Readnower RD Chavko M Adeeb S Conroy MD Pauly JR McCarron RM Increase in blood-brain barrier permeability, oxidative stress, and activated microglia in a rat model of blast-induced traumatic brain injury. J Neurosci Res (2010) 88(16):3530–9 10.1002/jnr.22510 20882564 PMC2965798

[20] Hernandez-Ontiveros DG Tajiri N Acosta S Giunta B Tan J Borlongan CV Microglia activation as a biomarker for traumatic brain injury. Front Neurol (2013) 4:30 10.3389/fneur.2013.00030 23531681 PMC3607801

[21] Woodcock T Morganti-Kossmann MC The role of markers of inflammation in traumatic brain injury. Front Neurol (2013) 4:18 10.3389/fneur.2013.00018 23459929 PMC3586682

[22] Balakathiresan N Bhomia M Chandran R Chavko M McCarron RM Maheshwari RK microRNA let-7i is a promising serum biomarker for blast-induced traumatic brain injury. J Neurotrauma (2012) 29(7):1379–87 10.1089/neu.2011.2146 22352906 PMC3335133

[23] Terrio H Brenner LA Ivins BJ Cho JM Helmick K Schwab K Traumatic brain injury screening: preliminary findings in a US army brigade combat team. J Head Trauma Rehabil (2009) 24(1):14–23 10.1097/HTR.0b013e31819581d8 19158592

[24] King PR Donnelly KT Donnelly JP Dunnam M Warner G Kittleson CJ Psychometric study of the neurobehavioral symptom inventory. J Rehabil Res Dev (2012) 49(6):879–88 10.1682/JRRD.2011.03.0051 23299259

[25] Wilkins KC Lang AJ Norman SB Synthesis of the psychometric properties of the PTSD checklist (PCL) military, civilian, and specific versions. Depress Anxiety (2011) 28(7):596–606 10.1002/da.20837 21681864 PMC3128669

[26] Trivedi MH Rush AJ Ibrahim HM Carmody TJ Biggs MM Suppes T The inventory of depressive symptomatology, clinician rating (IDS-C) and self-report (IDS-SR), and the quick inventory of depressive symptomatology, clinician rating (QIDS-C) and self-report (QIDS-SR) in public sector patients with mood disorders: a psychometric evaluation. Psychol Med (2004) 34(1):73–82 10.1002/mpr.285 14971628

[27] MacKenzie EJ Sacco WJ Luchter S Ditunno JF Staz CF Gruen GS Validating the functional capacity index as a measure of outcome following blunt multiple trauma. Qual Life Res (2002) 11(8):797–808 10.1023/A:1020820017658 12482163

[28] Morris DR Levenson CW Zinc in traumatic brain injury: from neuroprotection to neurotoxicity. Curr Opin Clin Nutr Metab Care (2013) 16(6):708–11 10.1097/MCO.0b013e328364f39c 23945221

[29] Papa L Lewis LM Silvestri S Falk JL Giordano P Brophy GM Serum levels of ubiquitin C-terminal hydrolase distinguish mild traumatic brain injury from trauma controls and are elevated in mild and moderate traumatic brain injury patients with intracranial lesions and neurosurgical intervention. J Trauma Acute Care Surg (2012) 72(5):1335–44 10.1097/TA.0b013e3182491e3d 22673263 PMC5516044

[30] Berger RP Hayes RL Richichi R Beers SR Wang KK Serum concentrations of ubiquitin C-terminal hydrolase-L1 and alphaII-spectrin breakdown product 145 kDa correlate with outcome after pediatric TBI. J Neurotrauma (2012) 29(1):162–7 10.1089/neu.2011.1989 22022780 PMC3253308

[31] Diaz-Arrastia R Wang KK Papa L Sorani MD Yue JK Puccio AM Acute biomarkers of traumatic brain injury: relationship between plasma levels of ubiquitin C-terminal hydrolase-L1 and glial fibrillary acidic protein. J Neurotrauma (2014) 31(1):19–25 10.1089/neu.2013.3040 23865516 PMC3880090

[32] Kobeissy F Mondello S Tümer N Toklu HZ Whidden MA Kirichenko N Assessing neuro-systemic & behavioral components in the pathophysiology of blast-related brain injury. Front Neurol (2013) 4:186 10.3389/fneur.2013.00186 24312074 PMC3836009

[33] Svetlov SI Prima V Glushakova O Svetlov A Kirk DR Gutierrez H Neuroglial and systemic mechanisms of pathological responses in rat models of primary blast overpressure compared to “composite” blast. Front Neurol (2012) 3:15 10.3389/fneur.2012.00015 22403567 PMC3275793

[34] Svetlov SI Prima V Kirk DR Gutierrez H Curley KC Hayes RL Morphologic and biochemical characterization of brain injury in a model of controlled blast overpressure exposure. J Trauma (2010) 69(4):795–804 10.1097/TA.0b013e3181bbd885 20215974

[35] Yokobori S Gajavelli S Mondello S Mo-Seaney J Bramlett HM Dietrich WD Neuroprotective effect of preoperatively induced mild hypothermia as determined by biomarkers and histopathological estimation in a rat subdural hematoma decompression model. J Neurosurg (2013) 118(2):370–80 10.3171/2012.10.JNS12725 23140154 PMC4968198

[36] Bilguvar K Tyagi NK Ozkara C Tuysuz B Bakircioglu M Choi M Recessive loss of function of the neuronal ubiquitin hydrolase UCHL1 leads to early-onset progressive neurodegeneration. Proc Natl Acad Sci U S A (2013) 110(9):3489–94 10.1073/pnas.1222732110 23359680 PMC3587195

[37] Sang Q Kim MH Kumar S Bye N Morganti-Kossman MC Gunnersen J Nedd4-WW domain-binding protein 5 (Ndfip1) is associated with neuronal survival after acute cortical brain injury. J Neurosci (2006) 26(27):7234–44 10.1523/JNEUROSCI.1398-06.2006 16822981 PMC6673957

[38] Yao XL Liu J Lee E Ling GS McCabe JT Cullin 5 gene expression in the rat cerebral cortex and hippocampus following traumatic brain injury (TBI). Neurosci Lett (2006) 409(1):65–9 10.1016/j.neulet.2006.09.015 17010517

[39] Wan C Chen J Hu B Zou H Li A Guo A Downregulation of UBE2Q1 is associated with neuronal apoptosis in rat brain cortex following traumatic brain injury. J Neurosci Res (2014) 92(1):1–12 10.1002/jnr.23305 24166684

[40] Sun KJ Zhu L Wang HD Ji XJ Pan H Chen M Zinc as mediator of ubiquitin conjugation following traumatic brain injury. Brain Res (2013) 1506:132–41 10.1016/j.brainres.2013.02.011 23419896

[41] Kotepui M Thawornkuno C Chavalitshewinkoon-Petmitr P Punyarit P Petmitr S Quantitative real-time RT-PCR of ITGA7, SVEP1, TNS1, LPHN3, SEMA3G, KLB and MMP13 mRNA expression in breast cancer. Asian Pac J Cancer Prev (2012) 13(11):5879–82 10.7314/APJCP.2012.13.11.5879 23317273

[42] Ferreira MA Matheson MC Tang CS Granell R Ang W Hui J Genome-wide association analysis identifies 11 risk variants associated with the asthma with hay fever phenotype. J Allergy Clin Immunol (2014) 133(6):1564–71 10.1016/j.jaci.2013.10.030 24388013 PMC4280183

[43] Chen YH Keller JJ Kang JH Lin HC Association between traumatic brain injury and the subsequent risk of brain cancer. J Neurotrauma (2012) 29(7):1328–33 10.1089/neu.2011.2235 22320191

[44] Liu B Neufeld AH Activation of epidermal growth factor receptors in astrocytes: from development to neural injury. J Neurosci Res (2007) 85(16):3523–9 10.1002/jnr.21364 17526018

[45] Liu B Neufeld AH Activation of epidermal growth factor receptor causes astrocytes to form cribriform structures. Glia (2004) 46(2):153–68 10.1002/glia.10358 15042583

[46] Tado M Mori T Fukushima M Oshima H Maeda T Yoshino A Increased expression of vascular endothelial growth factor attenuates contusion necrosis without influencing contusion edema after traumatic brain injury in rats. J Neurotrauma (2014) 31(7):691–8 10.1089/neu.2013.2940 24294928

[47] Yüksel H Yavuz Ö Is M Çomunoglu N Üzüm G Akyüz F Simvastatin reduces VEGF and NO levels in acute stages of experimental traumatic brain injury. Neurol Sci (2013) 34(11):1941–6 10.1007/s10072-013-1411-z 23543392

[48] Siddiq I Park E Liu E Spratt SK Surosky R Lee G Treatment of traumatic brain injury using zinc-finger protein gene therapy targeting VEGF-A. J Neurotrauma (2012) 29(17):2647–59 10.1089/neu.2012.2444 23016562

[49] Hanissian SH Teng B Akbar U Janjetovic Z Zhou Q Duntsch C Regulation of myeloid leukemia factor-1 interacting protein (MLF1IP) expression in glioblastoma. Brain Res (2005) 1047(1):56–64 10.1016/j.brainres.2005.04.017 15893739

[50] Vigodner M Shrivastava V Gutstein LE Schneider J Nieves E Goldstein M Localization and identification of sumoylated proteins in human sperm: excessive sumoylation is a marker of defective spermatozoa. Hum Reprod (2013) 28(1):210–23 10.1093/humrep/des317 23077236 PMC3522414

[51] Gill J Lee H Barr T Baxter T Heinzelmann M Rak H Lower health related quality of life in U.S. military personnel is associated with service-related disorders and inflammation. Psychiatry Res (2014) 216(1):116–22 10.1016/j.psychres.2014.01.046 24559851

[52] Jerg-Bretzke L Walter S Limbrecht-Ecklundt K Traue HC Emotional ambivalence and post-traumatic stress disorder (PTSD) in soldiers during military operations. Psychosoc Med (2013) 10:Doc03 10.3205/psm000093 23798980 PMC3687243

[53] Caldeira MV Salazar IL Curcio M Canzoniero LM Duarte CB Role of the ubiquitin-proteasome system in brain ischemia: friend or foe? Prog Neurobiol (2014) 112:50–69 10.1016/j.pneurobio.2013.10.003 24157661

[54] Reynolds JJ Stewart GS A nervous predisposition to unrepaired DNA double strand breaks. DNA Repair (Amst) (2013) 12(8):588–99 10.1016/j.dnarep.2013.04.011 23684796

